# Effect of semen and seminal amyloid on vaginal transmission of simian immunodeficiency virus

**DOI:** 10.1186/1742-4690-10-148

**Published:** 2013-12-05

**Authors:** Jan Münch, Ulrike Sauermann, Maral Yolamanova, Katharina Raue, Christiane Stahl-Hennig, Frank Kirchhoff

**Affiliations:** 1 Institute of Molecular Virology, Ulm University Medical Center, Meyerhofstrasse 1, 89081 Ulm, Germany; 2 Ulm Peptide Pharmaceuticals, Ulm University Medical Center, Meyerhofstrasse 1, 89081 Ulm, Germany; 3 German Primate Centre, 37077 Göttingen, Germany

**Keywords:** Semen-mediated enhancement of viral infection, SEVI, Amyloid, SIV, Transmission, Rhesus macaque

## Abstract

**Background:**

Semen and semen-derived amyloid fibrils boost HIV infection *in vitro* but their impact on sexual virus transmission *in vivo* is unknown. Here, we examined the effect of seminal plasma (SP) and semen-derived enhancer of virus infection (SEVI) on vaginal virus transmission in the SIV/rhesus macaque (Macacca mulatta) model.

**Results:**

A total of 18 non-synchronized female rhesus macaques (six per group) were exposed intra-vaginally to increasing doses of the pathogenic SIVmac239 molecular clone in the presence or absence of SEVI and SP. Establishment of productive virus infection was assessed by measuring plasma viral RNA loads at weekly intervals. We found that the first infections occurred at lower viral doses in the presence of SP and SEVI compared to the control group. Furthermore, the average peak viral loads during acute infection were about 6-fold higher after exposure to SP- and SEVI-treated virus. Overall infection rates after a total of 27 intra-vaginal exposures to increasing doses of SIV, however, were similar in the absence (4 of 6 animals) and presence of SP (5 of 6), or SEVI (4 of 6). Furthermore, the infectious viral doses required for infection varied considerably and did not differ significantly between these three groups.

**Conclusions:**

Semen and SEVI did not have drastic effects on vaginal SIV transmission in the present experimental setting but may facilitate spreading of virus infection after exposure to low viral doses that most closely approximate the *in vivo* situation.

## Background

Despite global efforts to limit the expansion of the AIDS pandemic, HIV-1 still causes about 2.3 million new infections each year. Most of these HIV-1 transmissions result from vaginal exposure to virus-containing semen during sexual intercourse [1-3]. Despite this dramatic spread of HIV-1, however, the efficiency of male-to-female intra-vaginal transmission is surprisingly low, with only about 1 event per 200 to 10,000 coital acts [4,5]. Thus, the poor transmissibility of HIV-1 clearly restricts the spread of the AIDS pandemic.

In addition to viral loads, the type of sexual practice, and the presence of other sexually transmitted diseases, factors modulating the infectiousness of HIV-1 in genital fluids may play a key role in the efficacy of sexual transmission of HIV-1 [6-8]. Multiple studies reported that semen boosts HIV-1 infection *in vitro*[9-17]. This enhancing effect correlates with the levels of amyloidogenic fragments of prostatic acid phosphatase and semenogelins that are abundant in human semen [12,13]. These semen-derived peptides rapidly form amyloid fibrils that facilitate virion attachment and may increase the infectiousness of HIV-1 in *in vitro* infection assays by several orders of magnitude [10-12]. Several agents that block this enhancing activity have been reported [14-18] and related fibril-forming peptides were developed for efficient lentiviral gene delivery [19]. Although semen is the main vector for the spread of HIV-1, its effect on sexual virus transmission *in vivo* is currently poorly understood.

Here, we used the SIV rhesus macaque non-human primate model [20,21] to examine possible effects of semen and semen-derived amyloid fibrils on vaginal virus transmission. A total of 18 rhesus macaques (six per group) were exposed intra-vaginally to increasing doses of the pathogenic SIVmac239 molecular clone in the presence or absence of SEVI and seminal plasma. Productive virus infection was assessed by measuring plasma viral RNA loads at weekly intervals.

## Results and discussion

**Figure 1:**
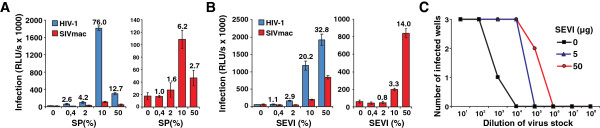
**SP and SEVI enhance SIVmac less effectively than HIV-1 infection. (A, B)** Effect of the indicated doses of SP **(A)** and SEVI **(B)** on infection of TZM-bl cells by HIV-1 NL4-3 (blue) or SIVmac239 (red). The left panels show infection by both viruses and the right panels SIVmac239 only. **(C)** Limiting dilution analysis of SIVmac239. CEM-M7 cells were infected in triplicate with 10-fold dilutions of the SIVmac239 virus stock in the presence of the indicated concentrations of SEVI. Indicated is the number of cultures that became productively infected.

To be able to use the same reagents throughout the entire *in vivo* study, we first generated large quantities of SIVmac stocks and SEVI solutions and collected pooled SP from healthy human donors. To ensure the efficacy of these reagents, we next examined the effect of SEVI and SP on the infectiousness of SIVmac and control HIV-1 stocks *in vitro*. We found that SP and SEVI enhanced infection by both HIV-1 and SIVmac, although the effects on the latter were substantially weaker (Figure 1A and B). SP was most effective at 10% (Figure 1A) since 50% begins to cause cytotoxic effects [10,13]. In contrast, the enhancing effect of SEVI did not saturate (Figure 1B). In limiting dilution infection assays, performed as previously reported [10], treatment with SEVI enhanced the TCID_50_ of SIVmac up to 100-fold (Figure 1C), which is about two orders of magnitude lower than previously observed for HIV-1 [10].

**Figure 2:**
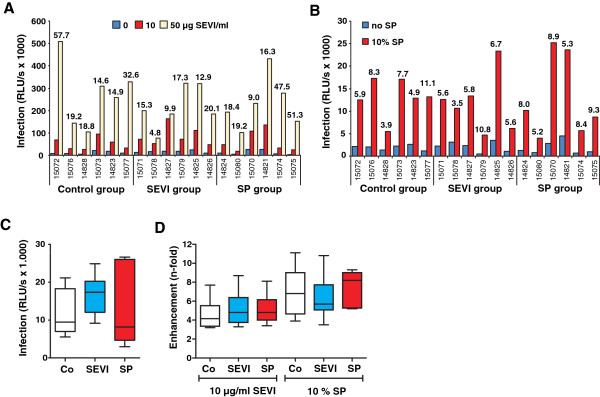
**Susceptibility of PBMCs derived from macaques assigned to the *in vivo *study to SIVmac239 infection and to SP- or SEVI-mediated enhancement of infection. (A, B)** Effect of SEVI **(A)** and SP **(B)** on SIVmac239 infection of PBMCs derived from the 18 animals assigned to the *in vivo* study. PHA/IL-2 stimulated cells were infected with SIVmac239 encoding a luciferase reporter gene [22]. Numbers above the bars indicate n-fold infectivity enhancement observed in the presence of SP or SEVI compared to those measured in their absence. RLU/s: relative light units per second. **(C, D)** PBMCs were grouped based on their belonging to the control (co), SEVI or SP groups in the subsequent *in vivo* study. The box plots in C show the mean infection rates and 25th and 75th percentiles measured in the absence of SP and SEVI. The box plots in **(D)** show the n-fold enhancement of SIVmac239 infection of PBMCs derived from the three groups of macaques in the presence of SEVI (10 μg/ml) or SP (10% v/v).

Since the effects of semen and SEVI on lentiviral infection have thus far almost exclusively been examined in human-derived cells [9-17], we next analyzed whether they can be confirmed in primary monkey cells. To assess possible differences in the susceptibility to SIV infection and the enhancing effects of SEVI or SP, we utilized PBMCs derived from all 18 animals assigned to the *in vivo* vaginal challenge study. We found that SEVI increased SIVmac239 infection of rhesus macaque PBMCs between 4.8- and 57.7-fold (Figure 2A), while SP-mediated enhancement ranged from 3.9- to 11.1-fold (Figure 2B). Notably, the absolute levels of SIVmac infection and the magnitudes of SEVI- and SP-mediated enhancement did not differ significantly between PBMCs derived from the three treatment groups of animals (Figure 2C and D).

**Figure 3:**
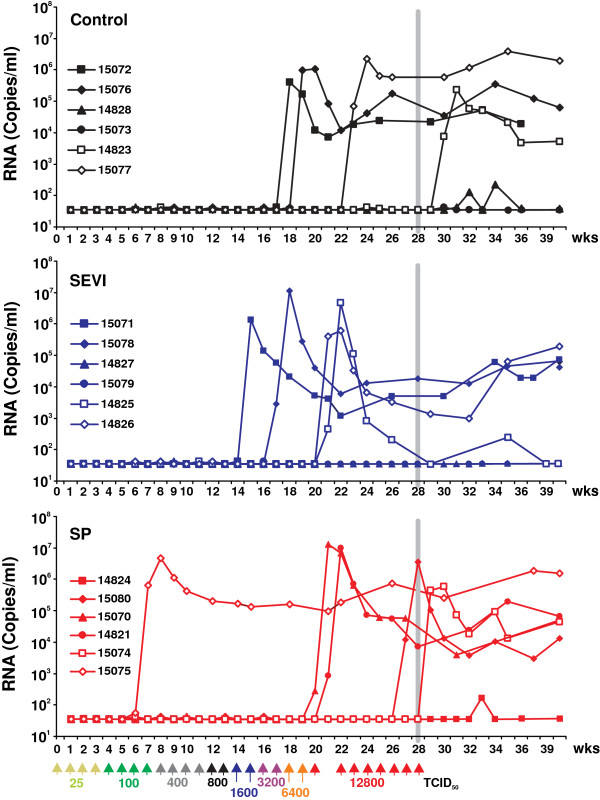
**SIV viral RNA loads in plasma of macaques after vaginal exposure to SIVmac239 in the absence (control) or presence of SEVI or SP.** The vertical gray line indicates the time point of the last virus inoculation. The detection limit was 40 viral RNA copies per ml plasma.

Our results showed that SEVI and SP enhance HIV-1 infection substantially more efficiently than infection by SIVmac. Nonetheless, the impact on SIVmac infection was readily detectable and highly significant. Thus, we decided to proceed with the *in vivo* study, although the effects in the SIV/macaque model may not fully reflect the impact of semen and SEVI in vaginal transmission of HIV-1. To closely mimic the situation during sexual virus transmission we did not synchronize the menstrual cycle of the female macaques or treat them with agents to induce thinning of the vaginal layer. The animals were exposed to up to 27 weekly non-traumatic vaginal challenges with gradually increasing doses of virus stocks that were either mixed with solutions of SEVI or SP or PBS (Figure 3). The final concentrations were 35 μg/ml of SEVI and 90% (v/v) of SP, the former was the yield of amyloidogenic PAP fragments from human semen [10], while the latter approximates the 100% of semen that is transferred during sexual intercourse. We used human semen because the challenges required a total of ~300 ml of this body fluid, a quantity which cannot be obtained from macaques. Furthermore, we felt that examination of the genuine vector of sexual transmission of HIV-1 in humans may have higher relevance for the spread of the AIDS pandemic than utilization of semen from a monkey species that is not a natural host of primate lentiviruses. However, vaginal exposure of macaques to human semen may affect their susceptibility to SIV infection by inducing immune reactions to the foreign antigens and local inflammation. Both enhancing effects due to the recruitment of activated viral target cells to the sites of virus exposure and protective effects due to the induction of local innate immune responses are conceivable. Clearly, the induction of immune responses to human antigens is a potential confounding factor. Notably, however, insemination is also associated with the induction of immunological processes and lymphocyte activation in humans [23-25]. Thus, some effects induced by vaginal exposure of macaques to human semen may reflect the events during sexual transmission of HIV-1 in humans.

**Figure 4:**
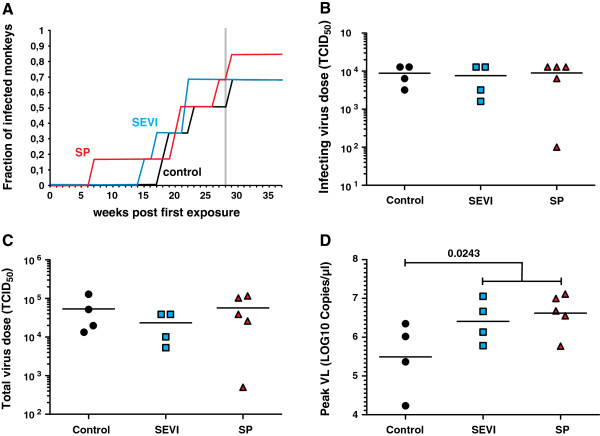
**Infection of rhesus macaques after vaginal exposure to SP- and SEVI-treated SIVmac. (A)** Proportion of infected animals in the three treatment groups as a function of the number of weekly vaginal exposures. **(B)** Administered viral dose (TCID_50_) leading to systemic infection and **(C)** total viral dose (cumulative TCID_50_) received by the animals until they became systemically infected. Two of the six macaques in the control and SEVI groups and one animal in the SP group are not shown since they remained uninfected after final virus exposure. **(D)** Peak viral loads during acute infection in the control, SEVI and SP groups.

The susceptibility of the animals to SIVmac infection after vaginal exposure varied considerably within each treatment group. One macaque (15075) in the SP group already became infected after the second exposure to 100 TCID_50_ (Figure 3). This animal showed signs of a mild infection of the respiratory tract at the week after productive SIV infection. However, there were no hints of inflammatory bacterial or viral genital infections. Thus, there were no obvious reasons for an enhanced susceptibility of animal 15075 to SIV infection. In strict contrast, five of the 18 animals (two in the control and SEVI groups and one in the SP group) remained uninfected even after eight weekly exposures to 12,800 TCID_50_ (Figures 3 and 4A). One animal in the SEVI group (14825) showed high levels of viremia during acute infection but subsequently efficiently controlled virus replication (Figure 3). Furthermore, one macaque (14828) assigned to the group of uninfected animals showed viral blips after the last virus exposure but did not become systemically infected (Figure 3). On average, the doses required for vaginal SIVmac infection did not differ significantly between the three treatment groups (Figures 4B and C). However, the first infections occurred at lower doses in the SP (100 TCID_50_) and SEVI (1,600 TCID_50_) groups than in the control (3,200 TCID_50_) group (Figures 3, 4A). Furthermore, the peak viral loads during acute infection were on average about 6-fold higher in animals that received SEVI and SP treated virus stocks (Figure 4D). This difference reached significance when the SEVI/SP groups were combined (p = 0.0243). The significance of these differences in VLs and the underlying mechanisms remain to be determined. It is tempting to speculate that weekly exposures to semen and seminal fibrils may enhance local inflammation [24-27] and that the resulting increase of activated viral target cells may facilitate initial virus spread. It has also been reported, however, that seminal fluid has immunosuppressive activity [28]. Thus, reduced innate immune control provides an alternative explanation for higher peak VLs after treatment with seminal plasma.

Our finding that semen may promote virus infection and spread after exposure to very low viral doses is in agreement with the results of two earlier studies that also reported increased vaginal SIV infection of macaques in the presence of semen, but only under conditions of low viral inoculums [29,30]. Finally, we found that polymorphisms in TRIM5α reported to affect the susceptibility to SIV infection [31] had no significant impact on the acquisition of SIVmac in the present study (Additional file 1: Figure S1). This is in agreement with the previous finding that the macaque adapted SIVmac239 strain is resistant against both wild-type and variant TRIM-5α alleles [31].

**Figure 5:**
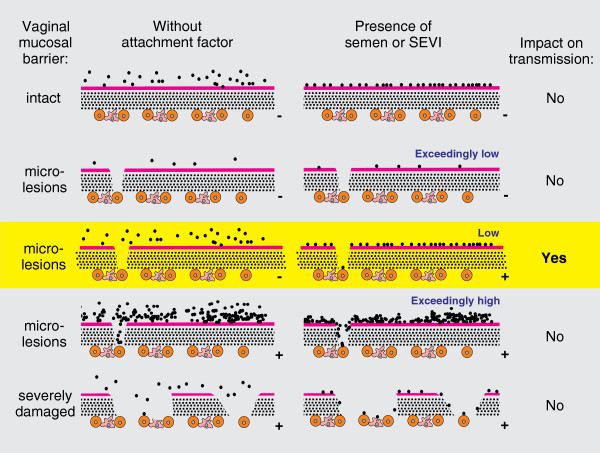
**Schematic outline of the possible roles of viral dose and the presence of microlesions in the effect of seminal attachment factors on sexual transmission of SIV and HIV-1.** Viral particles are indicated as black dots, mucus as pink line and the multi-layered vaginal epithelium by dashed lines. Infection of viral target T cells is indicated by the “+” symbol. Please note that this simplified model needs to be challenged in experimental studies.

Due to the limited number of animals and their high variability in susceptibility to vaginal SIV infection, our study provides only very preliminary insights into the role of semen and SEVI in sexual transmission of HIV-1. Our finding that the effects of SP and SEVI are not as striking as in cell culture systems did not come as a surprise since attachment factors that increase the stickiness of viral particles may only promote intra-vaginal virus transmission in the presence of micro-lesions in the vaginal mucosal layer and at low viral doses (schematically outlined in Figure 5). In agreement with the findings of previous studies [29,30], our results suggest that semen may increase the risk of vaginal transmission after exposure to low viral loads. Notably, even the repeated low dose challenge model utilizes viral quantities that are substantially higher than those transferred in humans because they usually achieve virus infection after about 10 to 20 challenges, whereas about 200 to 10,000 exposures are required for vaginal virus transmission by sexual intercourse. Furthermore, the effects of SEVI and SP on SIV infection *in vitro* were substantially weaker than those observed on HIV-1. Thus, the results of the present study may underestimate the effect of semen on the efficiency of HIV-1 transmission by sexual intercourse.

## Conclusions

In summary, our results show that SP and SEVI did not have drastic effects on vaginal SIV transmission in the present experimental settings, using non-synchronized macaques and increasing viral doses. However, SP and SEVI may facilitate spreading of virus infection after exposure to low viral doses that most closely approximate the *in vivo* situation. This study provides hints for the design of future animal studies of the effects of semen on the efficacy of sexual transmission of HIV-1. The effects of SP and SEVI on SIVmac239 infection *in vitro* were relatively weak compared to those previously observed for HIV-1. Notably, SIVmac239 was adapted to macaques through intravenous injections [32]. Thus, this molecular clone of SIV may not be well suited for studies on virus transmission via the genital mucosa. Currently, we are examining whether chimeric viral constructs (SHIVs) containing the HIV-1 Env glycoprotein may better reflect the full magnitude of the enhancing effects of semen and SEVI observed on HIV-1 infection. Furthermore, it is known that the phase of the menstrual cycle and different levels of vaginal inflammation, affect the susceptibility to HIV-1 and SIV infection [29,33,34]. Thus, consideration of the levels of pre-existing genital inflammation and/or rectal instead of vaginal exposure may deliver significant results with a reasonable number of animals. It would also circumvent biases due to menstrual cycle asynchrony, while the higher susceptibility associated with the presence of a single columnar layer of the rectal epithelium would reduce variations between donors. Such studies seem highly warranted since a better understanding of the role of semen and semen-derived amyloid fibrils in the spread of HIV-1 may be essential for the development of effective microbicides and vaccines.

## Methods

### Seminal plasma

Semen samples were collected at the “Kinderwunschzentrum” Ulm (Germany) from healthy individuals with informed consent. Pooled semen was generated from samples derived from > 20 individual donors. All ejaculates were allowed to liquefy for 30 min. SP represents the cell free supernatant of semen pelleted for 5 min at 10.000 rpm. In all experiments, aliquots were rapidly thawed and analyzed immediately.

### Generation of SEVI

PAP248-286 was produced by standard Fmoc solid-phase peptide synthesis, purified by preparative RP HPLC and analyzed by HPLC and MS. Lyophilized synthetic peptides were suspended in serum-free DMEM at concentrations of 5 to 10 mg/ml. Fibril formation was induced by overnight agitation at 37 °C and 1400 rpm using an Eppendorf Thermomixer and verified by Congo Red staining or electron microscopy.

### Virus stocks and infectivity

Virus stocks of HIV-1 NL4-3 and SIVmac239 were generated by transient transfection of 293 T cells as described [10].

### Effect of SP and SEVI on virus infection *in vitro*

The effect of SP and SEVI on HIV-1 and SIVmac infection and limiting dilution infection analyses were performed using adherent TZM-bl reporter cells or CEM-M7 cells as described previously [10,13].

### Animals

The animals assigned to this study were mature female rhesus monkeys (Macaca mulatta) of Chinese origin. These rhesus macaques were housed at the German Primate Center in accordance with the German Animal Welfare Act and in compliance with the European Union guidelines on the use of nonhuman primates for biomedical research. The study was approved by an ethics committee authorized by the Lower Saxony State Office for Consumer Protection and Food Safety.

### Vaginal exposure

To examine possible effects of SP and SEVI on SIV transmission, rhesus macaques were exposed vaginally once a week for up to 28 weeks to the SIVmac239 molecular clone except for a gap between week 20 and 22. The viral application procedure had been described before [35]. The intra-vaginal viral exposures were performed using escalating doses, i.e. with 25 TCID_50_ for the first four applications, 100 TCID_50_ for the following four challenges, and 400 TCID_50_ for another four exposures. Thereafter, doses were doubled after every second week eventually reaching 12.800 TCID_50_ for the final eight inoculations (see also Figure 3). Challenges were stopped 1 week after viral RNA became detectable in plasma with levels >100 copy equivalents/ml, indicating systemic infection. To prepare the inocula, virus stocks were diluted to a concentration 10-fold above the desired dose and either diluted in PBS buffer only (control group), a SEVI dilution to achieve a final concentration of 35 μg/ml (SEVI group) or undiluted SP (90% v/v; SP group). Virus exposures were done by non-traumatic inoculation of 0.25 ml of untreated, SP- or SEVI-treated virus into the vaginal tract. All experiments were done under highly controlled conditions by the same personnel using the same virus stock and inoculum preparation procedure.

### Viral RNA loads

Viral RNA copies were quantified in purified SIV RNA from plasma using TaqMan-based real-time PCR on an ABI-Prism 7500 sequence detection system (Applied Biosystems) as described [36].

#### Detection of TRIM5 polymorphisms

RNA was isolated from PBMCs using Qiagen RNAeasy Plus Minikit. RNA was reverse transcribed using Quantitect Reverse Transcription Kit (Qiagen, Germany). Trim5α-sequences were amplified by PCR using primers Trim5-for 5′-AGTGGAGAAGCTGCTATGGCT −3′and Trim5-rev 5′-ATGGACAAGAGGTGCTGTACAC −3′. Amplified PCR fragments were cloned into pJET1.2 (CloneJET PCR cloning kit, Fermentas). DNA-sequences of up to 5 clones in homozygous macaques were determined using Big Dye Cycle Sequencing. Amplicons were analyzed on an ABI 3130xL Genetic Analyser (Applied Biosystems). Sequence comparison was done with the freely available program Bioedit. TRIMcyp detection was performed by analysis of PCR fragment length polymorphism essentially as described [31].

#### Data analysis

The PRISM package version 5.0 (Abacus Concepts, Berkeley, CA) was used for all statistical calculations.

## Abbreviations

HIV-1: Human immunodeficiency virus type-1; AIDS: Acquired immunodeficiency syndrome; SEVI: Semen derived enhancer of viral infection; SIVmac: Simian immunodeficiency virus of rhesus macaques; SP: Seminal plasma; PBS: Phosphate buffered saline; TCID50: Median tissue culture infective dose; RP-HPLC: Reversed-phase high-performance liquid chromatography; MS: Mass Spectrometry.

## Competing interests

The authors declare they have no competing interests.

## Authors’ contributions

JM and YM performed *in vitro* experiments; US, KR and CSH performed animal experiments; JM, CSH and FK conceived and designed the study; FK wrote the paper. All authors have read and approved the final manuscript.

## Supplementary Material

Additional file 1: Figure S1 Susceptibility of rhesus macaques differing in their TRIM5 gene to infection by SIVmac239 after vaginal exposure. Animals that became infected after vaginal virus exposure were grouped based on the presence of homozygosity for TRIM5TFP allele (Wt, n = 5 of 7) or TRIM5∆∆Q (Del, n = 3 of 4) or heterozygous (Het, n = 4 of 6). TRIMCypA was absent in the macaques. Given are the numbers of animals that became infected out of the total number of macaques with the respective TRIM5 genotype. For one infected macaque the genotype could not be unambiguously determined. (A) Viral dose (TCID50) at the week before the animals became systemically infected. (B) Total viral dose (cumulative TCID50) inoculated into the animals until they became infected. Click here for file
