# Supplementary material for: Effect of semen and seminal amyloid on vaginal transmission of simian immunodeficiency virus
Source: Retrovirology. 2013 Dec 5;10:148. doi: 10.1186/1742-4690-10-148 (PMC4029343; doi:10.1186/1742-4690-10-148)
Supplement: Additional file 1: Figure S1 — Susceptibility of rhesus macaques differing in their TRIM5 gene to infection by SIVmac239 after vaginal exposure. Animals that became infected after vaginal virus exposure were grouped based on the presence of homozygosity for TRIM5TFP allele (Wt, n = 5 of 7) or TRIM5∆∆Q (Del, n = 3 of 4) or heterozygous (Het, n = 4 of 6). TRIMCypA was absent in the macaques. Given are the numbers of animals that became infected out of the total number of macaques with the respective TRIM5 genotype. For one infected macaque the genotype could not be unambiguously determined. (A) Viral dose (TCID50) at the week before the animals became systemically infected. (B) Total viral dose (cumulative TCID50) inoculated into the animals until they became infected. [file 1742-4690-10-148-S1.pdf]

Effect of semen and seminal amyloid on vaginal transmission of simian immunodeficiency virus

Jan Münch, Ulrike Sauermann, Maral Yolamanova, Katharina Raue, Christiane Stahl-Hennig, and Frank Kirchhoff

Additional file

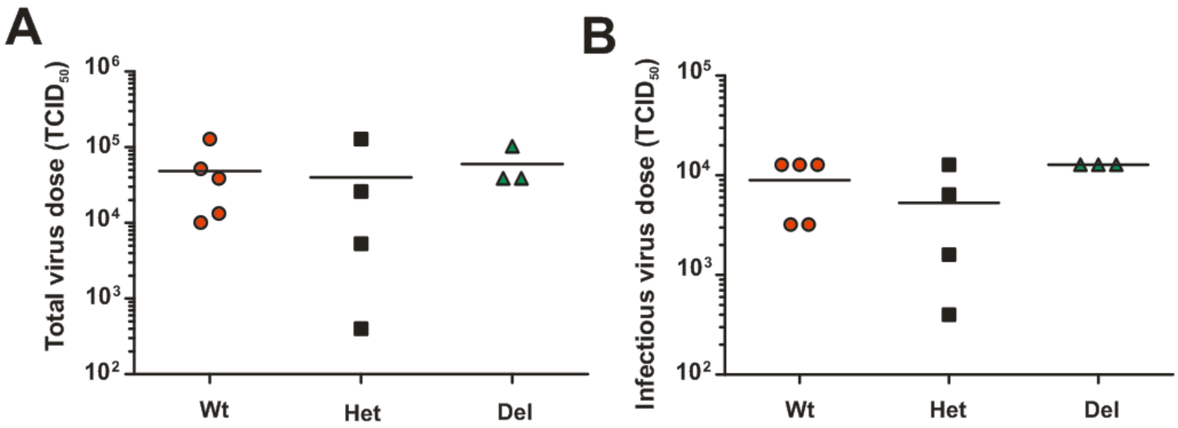

Figure S1. Susceptibility of rhesus macaques differing in their TRIM5 gene to infection by SIVmac239 after vaginal exposure. Animals that became infected after vaginal virus exposure were grouped based on the presence of homozygosity for *TRIM5*^{TFP} allele (Wt, n=5 of 7) or *TRIM5*$^{\Delta\Delta Q}$ (Del, n=3 of 4) or heterozygous (Het, n=4 of 6). *TRIM*^{CypA} was absent in the macaques. Given are the numbers of animals that became infected out of the total number of macaques with the respective *TRIM5* genotype. For one infected macaque the genotype could not be unambiguously determined. (A) Total viral dose (cumulative $TCID_{50}$) inoculated into the animals until they became infected and (B) viral dose ($TCID_{50}$) at the week before the animals became systemically infected.
